# Metabolomics Response for Drought Stress Tolerance in Chinese Wheat Genotypes (*Triticum aestivum*)

**DOI:** 10.3390/plants9040520

**Published:** 2020-04-17

**Authors:** Xiaoyang Guo, Zeyu Xin, Tiegang Yang, Xingli Ma, Yang Zhang, Zhiqiang Wang, Yongzhe Ren, Tongbao Lin

**Affiliations:** 1 College of Agronomy, Henan Agricultural University, Zhengzhou 450002, China; guoxiaoyang@stu.henau.edu.cn (X.G.); zeyuxin@henau.edu.cn (Z.X.); maxingli@henau.edu.cn (X.M.); wzq@henau.edu.cn (Z.W.); yzren@henau.edu.cn (Y.R.); 2 Industrial Crops Research Institute, Henan Academy of Agricultural Sciences, Zhengzhou 450002, China; yangtg@hnagri.org.cn; 3 Institute of Agricultural Resources and Regional Planning, Chinese Academy of Agricultural Sciences, Beijing 10081, China; zhangyang03@caas.cn

**Keywords:** untargeted approach, drought stress, metabolites, photosynthesis indices, wheat

## Abstract

Metabolomics is an effective biotechnological tool that can be used to attain comprehensive information on metabolites. In this study, the profiles of metabolites produced by wheat seedlings in response to drought stress were investigated using an untargeted approach with ultra-performance liquid chromatography-mass spectrometry (UPLC-MS) to determine various physiological processes related to drought tolerance from the cross between drought-tolerant genotype (HX10) and drought-sensitive genotype (YN211). The current study results showed that under drought stress, HX10 exhibited higher growth indices than YN211. After drought stress treatment, a series of phenolics accumulated higher in HX10 than in YN211, whereas the amount of thymine, a pyrimidine, is almost 13 folds of that in YN211. These metabolites, as well as high levels of different amino acids, alkaloids, organic acids, and flavonoids in the drought treated HX10 could help to explain its strong drought-tolerant capacity. The current study explored the understanding of the mechanisms involved in the drought response of wheat seedling; these metabolome data could also be used for potential QTL or GWAS studies to identify locus (loci) or gene(s) associated with these metabolic traits for the crop improvement.

## 1. Introduction

Wheat (*Triticum aestivum*) is one of the most widely cultivated cereal crops all over the world. In China, owing to its high nutritional content of carbohydrates, wheat has been recognized as a staple food for a long time [1]. However, in the process of wheat growth and development, it always suffers many abiotic stresses, such as drought, dry-hot wind, low temperature, water-logging, and so on [2].

Drought stress has profound negative impacts on plants at the morphological, physiological, and biochemical levels, with decreased photosynthesis [3], impaired cell elongation and division [4], and losses of cell turgor [5]. Drought stress also inhibits plants from further nutrient absorption and affects the gene expression, distribution, yield, and quality of crops [6,7,8]. In recent years, the losses of winter wheat grain yield induced by drought stress were increasing over all the wheat growing areas across the globe [9,10,11].

In nature, many plants have adapted to various abiotic stresses by using their enormous metabolic homeostasis to produce a large variety of primary and secondary metabolites. During abiotic stresses, plant metabolites and related pathways can be influenced [12,13]. In the case of drought stress, plant responses may involve a few important metabolic pathways, such as photosynthesis, sugar synthesis, tricarboxylic acid (TCA) cycle, glycolysis, and hormone synthesis [14,15,16]. Many metabolites such as fructose, sucrose, proline, and other amino acids, and betaine could contribute to drought stress tolerance [17,18,19].

Metabolomics has largely focused on the organic molecular compounds (metabolites) and the related biochemical changes found in or produced by organisms and their tissues and cells [20,21,22]. In plant researche, metabolomics is now considered a widespread significant biotechnological tool for different molecular biology studies. 

Metabolomics includes multifarious analytical approaches to identify organic molecular metabolomic components, for instance, metabolic fingerprinting, metabolite profiling and targeted analysis, gas chromatography-mass spectrometry (GC-MS), liquid chromatography-mass spectrometry (LC-MS), and nuclear magnetic resonance (NMR). These technologies can be employed to identify metabolomic components accurately [23,24,25]. Previous studies have focused on the drought responses and tolerance of plants by means of metabolomic technologies. For example, Bowne et al. used a targeted GC-MS approach to identify compounds that differ in three different genotypes of bread wheat characterized by different levels of tolerance under drought stress [26]. Using an LC-MS method, Alvarez et al. found increased proline, malate, p-coumarate, and caffeate and decreased ferulate in the maize xylem sap under water stress [27]. Charlton et al. found increased levels of c-amino butyrate (GABA), and myoinositol, malate, as well as proline, leucine, isoleucine, valine, threonine, and homoserine in pea leaves (*Pisum sativum L.*) under simulated field conditions, but noted that leucine and isoleucine were not elevated when grown in a greenhouse using NMR [28]. However, more metabolite secrets in the plant drought tolerance are still waiting to be revealed. Thus, the aim of this paper was to investigate the metabolites of the seedlings of two wheat genotypes with different levels of drought tolerance using ultra-performance liquid chromatography-mass spectrometry (UPLC-MS) to increase our understanding of drought-tolerant mechanisms of wheat.

## 2. Results

### 2.1. Physiological Responses to Drought Stress

**Figure 1:**
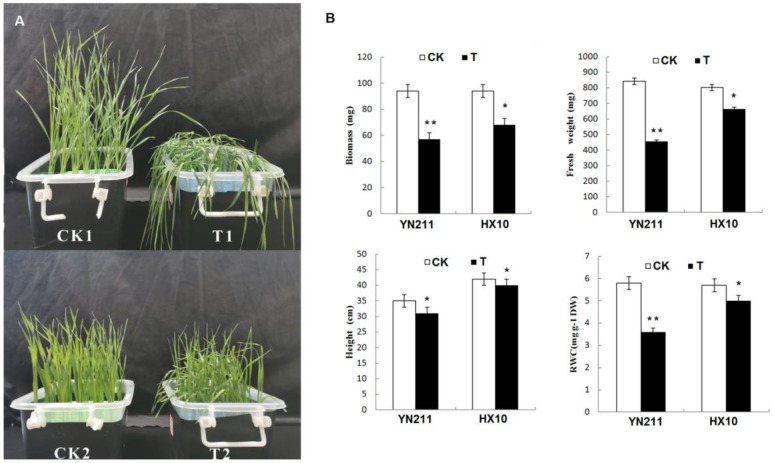
Growth performances of two wheat genotypes under control and drought stress conditions at the seedling stage. (**A**), the two wheat genotypes exhibited morphological differences after seven days treatment with 20% PEG. (**B**), growth parameters of two wheat genotypes. CK1:YN211 under normal conditions, T1: YN211 under drought treatment, CK2:HX10 under normal conditions, T2: HX10 under drought treatment. Asterisk (*) and double asterisk (**) indicate significant (*p* < 0.05) and highly significant (*p* < 0.01) differences between controls and treatments, respectively.

After treated by 20% poly-ethylene glycol (PEG) for seven days, the growth parameters of wheat seedlings were measured. The results showed that the drought stress significantly influenced the growth of wheat seedlings, especially for the drought-sensitive genotype YN211. As shown in Figure 1, both sensitive and tolerant cultivars showed retarded growth compared with the untreated control samples (CK) after seven days of treatment. For the YN211, biomass, fresh weight, and the relative water content (RWC) were markedly decreased. However, the influence of drought treatment on the growth of HX10 was limited. The seedling height, fresh weight, and the relative water content (RWC) of HX10 were only reduced a little compared with the CK samples.

### 2.2. Metabolite Profile under Drought Stress

#### 2.2.1. Principal Component Analysis of Metabolites Profiles

A total of 691 peaks were detected, of which 175 were identified as known metabolites, and the remaining peaks were unknown metabolites. The identified metabolites include amino acids, organic acids, sugars, polyamines, nitrogenous compounds, and polyphenols.

**Figure 2:**
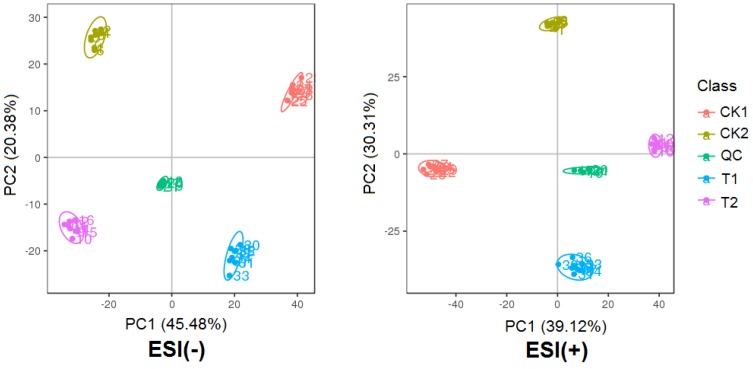
Principal component analysis (PCA) of metabolic profiles in leaves of HX10 and YN211 under control or drought stress (eight biological replicates). CK1:YN211 under normal conditions, T1: YN211 under drought treatment, CK2:HX10 under normal conditions, T2: HX10 under drought treatment.

Principle components analysis (PCA) was performed to evaluate the repeatability and stability of the metabolic profiles from the UPLC-MS and compare the similarities and differences among all samples. Figure 2 showed that for both the electron spray ionization (ESI) models, ESI (-) and ESI (+), samples including the quality control (QC) samples were clearly divided into five groups, and samples from the same genotypes and treatments were grouped together, demonstrating the high quality of the data. This result indicated that drought stress and wheat genotypes had a combinatory effect on metabolite variation of the samples, and both factors influenced metabolite variation significantly.

#### 2.2.2. Partial Least Square Discriminant Analysis of Metabolites Profiles

**Figure 3:**
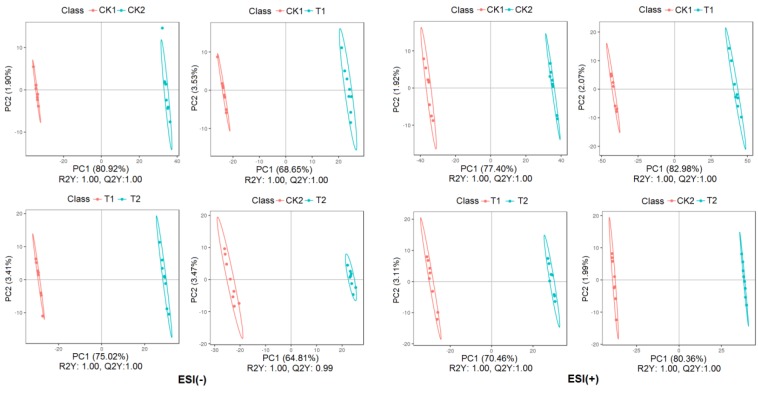
Partial least squares discriminant analysis (PLS-DA) of metabolic profiles in the leaves of HX10 and YN211 under control or drought stress (eight biological replicates). CK1:YN211 under normal conditions, T1: YN211 under drought treatment, CK2:HX10 under normal conditions, T2: HX10 under drought treatment.

Partial least square discriminant analysis (PLS-DA) was also conducted to compare the differences between groups CK1 vs. CK2, T1 vs. T2, CK1 vs. T1, CK2 vs. T2 in ESI (+) or ESI (-) model. Similar classification results with PCA analysis was observed from Figure 3. Samples gathered in a certain area according to the treatment or genotype. All R2Y and Q2Y data supported the predictive accuracy of the model.

The scores plot between PC1 and PC2 revealed two distinct groups associated with the drought and control samples seven days after drought stress (Figure 3), suggesting the clear distinction in the metabolite accumulation under normal conditions and drought stress treatment. Furthermore, the sensitive genotype (YN211) and tolerant genotype (HX10) samples were separated from each other under both drought and control conditions.

**Figure 4:**
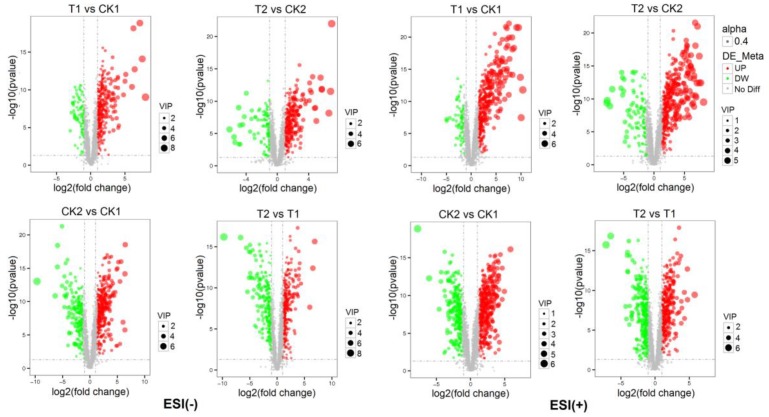
Volcanic plots of metabolites in wheat leaves of two genotypes under control or drought. The *x*-axis represents log2 value of fold change of each metabolite, *y*-axis represents the *p*-value of metabolites (log10 value), gray represents metabolites with no significant difference, red represents up-regulated metabolites, green represents down-regulated metabolites. CK1:YN211 under normal conditions, T1: YN211 under drought stress, CK2:HX10 under normal conditions, T2: HX10 under drought stress.

PLS-DA was also utilized to identify the important metabolites associated with the drought condition based on the VIP (Variable Importance in the Projection) score (VIP > 1.0, fold change (FC > 2.0 or FC < 0.5) and *p*-value < 0.05) (Table 1, Figure 4). In general, 2544 metabolites were detected in ESI (-) model. For sensitive genotype YN211, the concentrations of 464 metabolites were changed between T1 and CK1, including 366 increased and 98 reduced. For the drought-tolerant genotype HX10, the concentrations of 429 metabolites were changed between T2 and CK2, including 318 increased and 111 reduced. Six hundred ten metabolites showed obvious differences between the two genotypes, including 390 increased and 220 reduced (CK2 vs. CK1). Under drought treatment, 584 metabolites showed obvious differences between two genotypes, including 326 increased and 258 reduced (T2 vs. T1). A total of 3028 metabolites were detected in the ESI (+) model. For sensitive genotype YN211, the concentrations of 527 metabolites were changed between T1 and CK1, including 449 increased and 78 reduced. For the drought-tolerant genotype HX10, the concentrations of 499 metabolites were changed between T2 and CK2, including 397 increased and 102 reduced. Eight hundred eleven metabolites showed obvious differences between the two genotypes, including 523 increased and 288 reduced (CK2 vs. CK1). Under drought treatment, 797 metabolites showed obvious differences between two genotypes, including 425 increased and 372 reduced (T2 vs. T1).

#### 2.2.3. Dynamic Metabolic Changes of Drought-Tolerant Genotype and Drought-Sensitive Wheat Genotype under Normal Conditions or Drought Stress

Kruskal-Wallis ANOVA was used to compare the overall variation in metabolism associated with drought stress in different genotypes. Fourteen changed metabolites were identified in YN211 between T1 and CK1, including 12 up-regulated and two down-regulated, while 10 changed metabolites were identified in HX10, including four up-regulated and six down-regulated. The metabolites were different between two genotypes under normal conditions. Compared with HX10, there were 18 metabolites with a higher level and 24 with a lower level in the drought-sensitive YN211. Major metabolites were organic acids (caffeic acid, ascorbic acid, gentinic acid, syringic acid) and flavones (amygdalin, astragaloside, kaila anthocyanin, flavone of rehmannia mauritiana, baima glycoside, and arbutin).

**Figure 5:**
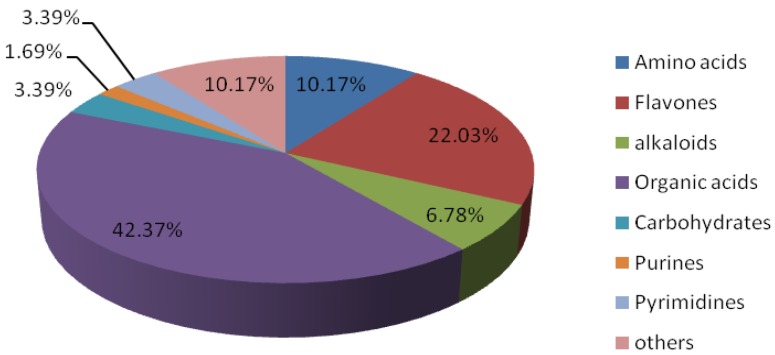
Classification of 56 differently accumulated metabolites between two wheat genotypes under drought stress.

Under drought treatment, mass spectrometry analysis of changed metabolites enabled the identification of 56 metabolites, including amino acids, organic acids, carbohydrates, alkaloids, flavones, purines, and pyrimidines (Table 2, Figure 5, Figure S1). There were 24 metabolites with a higher level in HX10, and 32 with a lower level, compared with YN211. A series of phenolics including homovanillic acid, gallic acid, vanillin, 3,4-dihydroxybenzaldehyde, 3-dimethylallyl-4-hydroxymandelic acid, alpha-phocaecholic acid, kynurenic acid, coniferyl aldehyde, vanillyl alcohol, pyridoxal, and picolinic acid accumulated more in HX10. Furthermore, the thymine, which is a pyrimidine accumulated highly in drought-tolerant genotype with almost 13-fold of the amount in YN211. Meanwhile, a purine, guanine, also accumulated more in HX10. Other metabolites with a higher level in HX10 were two flavonoids, dhurrin and genistein; the amino acids, L-cysteinylglycine and fructoselysine; alkaloids, dihydroxyindole and scopoletin; citric acid (organic acids), farney pyrophosphate, D-Ribulose 5-phosphate, and anti-zeatin. Metabolites with a low level in drought treated HX10 compared with YN211 mainly included amino acids, flavonoids, alkaloids, phenolics, and organic acids (Table 2).

**Figure 6:**
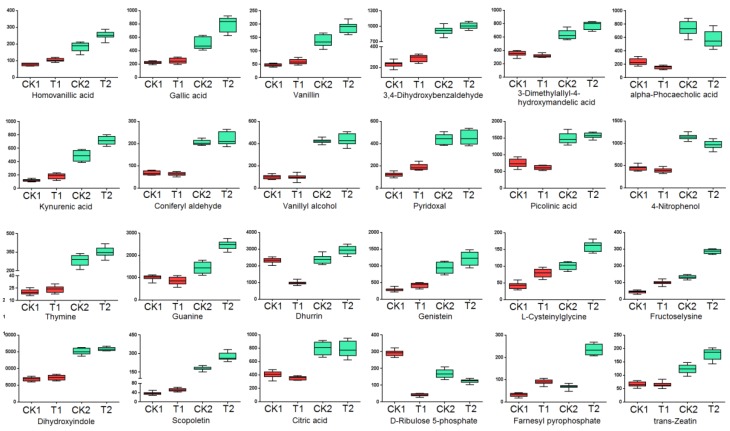
Relative contents of the 24 metabolites accumulated at a higher level in HX10 after drought treatment in all samples. CK1:YN211 under normal conditions, T1: YN211 under drought treatment, CK2:HX10 under normal conditions, T2: HX10 under drought treatment.

Analysis of the detailed levels of these different metabolites in all samples showed that all of the phenolics, as well as the thymine and guanine with a higher level in the drought-treated HX10, were also highly accumulated in the untreated HX10 (Figure 6), demonstrating that the high level of these metabolites in HX10 was a consistent feature rather than an environment induced feature. Similarly, the two amino acids enriched in drought treated HX10 were also showed higher level in untreated HX10. However, both of them were obviously induced after treatment in both genotypes. This expression pattern also occurred for farney pyrophosphate, which is the precursor of sesquiterpenes and triterpenes which includes the well-known drought-related hormone abscisic acid. The high level of farney pyrophosphate may indicate a high level of abscisic acid.

## 3. Discussion

In the comparison of different metabolites identified in two genotypes under drought stress, as much as 12 phenolics showed a higher level in HX10, accounting for half of the total 24 higher accumulated metabolites in this genotype. Phenolics are a group of important antioxidants, the production of phenolic compounds (or polyphenols) is one of the strategies used by plants to avoid the oxidative damage caused by drought [29]. It was reported that the level of phenolic compounds could be used as indicators of drought resistance in shrubs [30]. Moreover, studies have shown that the drought stress could increase the number of phenolic compounds in some plants, and some drought resistant genotype could produce more phenolic compounds with or without drought treatment [31,32]. The current study also indicated the critical role of phenolic compounds in the drought tolerance ability of HX10. It is known that drought stress exacerbates reactive oxygen species (ROS) production in plant cells, which causes oxidative damage such as membrane disruption, protein degradation, enzyme inactivation, and ironic imbalance [33,34,35]. Phenolics have been proved to be a group of important antioxidants, the production of phenolic compounds (or polyphenols) is one of the strategies used by plants to avoid the oxidative damage caused by drought stress [29,36,37]. Total phenolics content, total flavonoids content, anthocyanin content, and schaftoside content in wheat leaves were enhanced during drought treatment [38]. A previous study showed that the protective role of phenolic compounds is due to the special structure, e.g., hydroxyl group, double carbon bonds, and modifications like glycosylation, prenylation, and methylation [29,38]. All the 12 phenolic compounds identified in HX10 in the current study have hydroxyl group, double carbon bonds, or the methyl group. Thus, the highly accumulated phenolic compounds in HX10 might act as powerful antioxidants to protect the plant from drought stress caused oxidative damage. 

The current study revealed the significantly higher amount of thymine (a purine nucleotide) and guanine (a pyrimidine nucleotide) in the drought-tolerant genotype HX10. As shown in previous studies, the promotion of purine and pyrimidine nucleotide biosynthesis provided ATP energy through drought stress [39,40]. Furthermore, the purine degradation contributed to the protective responses such as synergistic activation of abscisic acid metabolism and accumulation of the cellular protectant proline to drought stress [41,42]. Besides, there were studies showing that drought stress could induce the increase of purine and pyrimidine by transcriptome or metabolomics [43,44]. Thus, the current study indicated that thymine and guanine might contribute to the drought tolerance of HX10 by producing energy and enhancing protective responses. 

Metabolomic analysis showed increased proline levels in leaves of drought-sensitive genotype YN211 subjected to drought, but not in HX10. Apart from proline, several other amino acids were clearly induced in YN211 but not in HX10. Many amino acids have been shown in response to the water/drought stress either at a higher or lower level [45,46]. Proline is one of the main osmolytes in plants and could accumulate in response to various abiotic stresses [47]. Besides acting as an excellent osmolyte, proline plays three major roles during stress, i.e., as a metal chelator, an antioxidative defense molecule, and a signaling molecule [47,48]. A recent investigation on bread wheat genotypes showed that proline content significantly increased under stress, but weakly or not significantly correlated with agronomic traits such as plant height under both optimal, and water-limited conditions [49]. In the current study, alghough the plant height was also not correlated with proline content. However, the proline did not elevate in drought-tolerant genotype HX10, indicating that there may be another mechanism for its tolerant capacity. The present study observed the increased amount of two special amino acids, L-cysteinylglycine, and fructoselysine, in the drought-tolerant genotype. In plants, reduced glutathione (GSH), which is a tripeptide constituted of glutamate, cysteine, and glycine, is considered the most important intracellular defense against ROS in plant cells among nonenzymetic antioxidants [50]. The presence of Cys in the chemical reactivity and higher water solubility of the thiol (-SH) group of GSH confers its biological properties and make it a crucial metabolite to perform multiple functions including growth, development and plant responses to drought stress [51,52]. Thus, the L-cysteinylglycine with Cys and thiol may also be considered with strong confer antioxidants property in drought responses in wheat. For fructoselysine, a previous study in Arabidopsis showed that oxidative stress led to increased protein glycation [53] and glucoselysine as well as fructoselysine were proved with protective antioxidative activity [54]. The fructoselysine in the drought-tolerant genotype may also take part in its drought tolerance as an antioxidant.

## 4. Materials and Methods 

### 4.1. Materials and Growth Conditions

Wheat genotype Hanxuan10 (HX10) and Yunong211 (YN211) were used in this study. Hanxuan10 is an important source in China with drought resistance which collected from Luoyang Academy of Agriculture and Forestry Sciences, Luoyang City, Henan Province, China. HX10 is widely grown in semi-arid areas under rain-fed conditions. YN211 is one of the drought-sensitive wheat genotypes grown in Henan Province, China. Both of HX10 and YN211 are hexaploid. HX10 was released in 1966. HX10 has excellent characteristics of drought-resistance, barren-resistance, and cold-resistance, which was grown widely in dryland wheat regions of North China. Its germplasm number is ZM009279. YN211 was cultivated by Henan Agricultural University in 2014, China, and cultured through breeding Yunong201//Yunong9234903/Baiyingdong. The germplasm number of YN211 is Yushenzhengzi2014004.

Formic acid was purchased from Waters Corporation (Waters Corporation, Milford, MA, USA). Acetonitrile and methanol were purchased from Fisher Scientific; Ultra-pure water was prepared using a Millipore Alpha-Q water purification system (Millipore Corporation, Bedford, MA, USA) [55]. 

The seeds of HX10 and YN211 were surface-sterilized with 70% alcohol and 0.1% HgCl for 5 min and 15 min, respectively. Then washed five times with distilled water and soaked in water for 12 h, and then incubated in water in the dark at 25 °C for three days to germinate. Seedlings were then cultivated in 1/2 Hoagland’s nutrient solution in a light incubator at 25 °C/22 °C with 16/8 h light/dark photoperiod and 65% relative humidity. Hoagland’s nutrient solution was changed every three days [56]. 

### 4.2. Treatments

At the two-leaf stage, YN211 and HX10 seedlings were subjected to drought stress induced by 20% (m/v) poly-ethylene glycol-6000 (PEG-6000; −0.975 MPa) and designated as T1 and T2, respectively. YN211 and HX10 seedlings grown under normal conditions were used as the controls and were designated as CK1 and CK2, respectively. The culture solutions were refilled twice a week. Leaf tissues were collected seven days after 20% PEG treatment. Fresh materials were used to measure the physiological indexes. The dried biomass was determined using samples dried at 70 °C overnight. The samples used to analyze the metabolite contents were frozen immediately in liquid nitrogen and stored at −80 °C before analysis. For physiological analysis, the treatments were repeated in triplicate, and for metabolomics analysis, the treatments were repeated eight times to control experimental variability [56]. All samples were collected, marked, and frozen immediately in liquid nitrogen for 20 min and stored at −80 °C before analysis.

### 4.3. Growth Parameters

After seven days of drought treatment, a series of growth parameters were recorded. Plant height was measured using a metric ruler. To determine the plant biomass, leaves were collected and washed by distilled water, then dried at 60 °C for 72 h, and the biomass was weighed using an electronic scale.

Leaf relative water content (RWC) was determined with fully expanded leaves after seven days of drought stress imposition [57]. Briefly, leaves were cut off and weighed (fresh weight, FW) and then soaked in water at room temperature for 24 h and then wiped down excess water with paper towels immediately before weighing (saturated weights, SW). The leaves were dried in an oven at 60 °C for 72 h and weighed as the dry weight (DW). Leaf RWC was calculated using the following formula: RWC (%) = (FW-DW)/(SW-DW) × 100.

### 4.4. Metabolite Extraction

Leaves (100 mg) were individually grounded with liquid nitrogen, and the homogenate was resuspended with prechilled 80% methanol (−20 °C) followed by good vortexing. The samples were incubated at −20 °C for 60 min and then were centrifuged at 14,000× *g*, 4 °C for 20 min. The supernatants were subsequently transferred to a fresh Eppendorf tube and spun in a vacuum concentrator until dry. The dried metabolite pellets were resuspended by 60% methanol and analyzed by LC-MS/MS. All samples were randomized to eliminate instrument errors. To monitor the stability and repeatability of the instrument analysis, quality control (QC) samples were prepared, while sample processing. The QC samples were equally mixing samples of experimental samples, which were used to balance the chromatographic-mass spectrometry system, monitor the state of LC-MS system performance, and to evaluate the stability of the system during the whole experiment process. The correlation value of the QC samples was positively related to the stability of the method [58]. If the distribution of the QC samples mixed together in the PCA analysis diagram, this would demonstrate that the correlation value of QC samples is high [59]. At the same time, blank samples were set up to remove background ions.

### 4.5. Chromatographic Separation

An untargeted approach with UHPLC-MS/MS analysis was used to identify metabolites associated with the alteration of wheat seedlings in response to drought stress. A Vanquish UHPLC system (Thermo Fisher Scientific, Bremen, Germany) fitted with Q-Exactive HF-X Orbitrap mass spectrometer (Thermo Fisher Scientific, Bremen, Germany) operating in the data-dependent acquisition (DDA) mode was used in the current study. 

Chromatographic separation was performed with a Vanquish UHPLC system (Thermo Fisher Scientific, Bremen, Germany). Samples were injected onto an Accucore HILIC column (100 × 2.1 mm, 2.6 μm) using a 16-min linear gradient at a flow rate of 0.3 mL/min. The eluents for the positive polarity mode were eluent A (0.1% formic acid (FA, Waters Corporation, Milford, USA) in 95% acetonitrile (ACN), 10 mM ammonium acetate) and eluent B (0.1% FA in 50% ACN, 10 mM ammonium acetate). The eluents for the negative polarity mode were eluent A (95% ACN, 10 mM ammonium acetate, pH 9.0) and eluent B (50% ACN, 10 mM ammonium acetate, pH 9.0). The solvent gradient was set as follows: 2% B, 1.5 min; 2–100% B, 12.0 min; 100% B, 14.0 min; 100–2% B, 14.1 min; 2% B, 16 min. The flow rate was 300 µL/min. The column temperature was set at 40 ℃ [60].

### 4.6. Mass Spectrometry

Detection of the compounds was performed using Q-Exactive HF-X Orbitrap Mass Spectrometer (Thermo Fisher Scientific, Bremen, Germany) operating in the data-dependent acquisition (DDA) mode. Q-Exactive HF-X mass spectrometer was operated in positive/negative polarity mode with a spray voltage of 3.2 kV, capillary temperature of 320 ℃, sheath gas flow rate of 35 arb and aux gas flow rate of 10 arb [60].

### 4.7. Data Analysis

The raw data files generated by UHPLC-MS/MS analysis were processed using the Compound Discoverer 3.0 (CD 3.0, Thermo Fisher Scientific, San Jose, CA, USA) to perform peak alignment, peak picking, and quantitation for each metabolite. The main parameters were set as follows: retention time tolerance, 0.2 minutes; actual mass tolerance, 5ppm; signal intensity tolerance, 30%; signal/noise ratio, 3; minimum intensity, 100,000. After that, peak intensities were normalized to the total spectral intensity. The normalized data were used to predict the molecular formula based on additive ions, molecular ion peaks, and fragment ions. Then peaks were matched with the mzCloud (https://www.mzcloud.org/) and ChemSpider (http://www.chemspider.com/) database to obtain the accurate qualitative and relative quantitative results. The resulting data were subjected to principal component analysis (PCA) and Partial Least Square Discriminant Analysis (PLS-DA) using the R program [60]. 

## 5. Conclusions

The present study compared the metabolites of the drought-sensitive genotype YN211 and drought-tolerant wheat genotype HX10 under drought treatment by untargeted analysis with UPLC-MS data. Under drought treatment, the growth of HX10 only showed slight retardation, especially in the seedling height, fresh weight, and RWC, compared with YN211. Fifty-six changed metabolites were identified between the two genotypes under drought /PEG 6000 treatment. In HX10, a series of phenolics accumulated at a higher level than those in YN211, and the amount of a pyrimidine-thymine is almost 13 folds of that in YN211. These metabolites, as well as high levels of amino acids, alkaloids, organic acids, and flavonoids, could help to explain the strong drought-tolerant capacity of HX10.

The current study provided important information on metabolites about the two wheat genotypes HX10 and YN211, which differ in drought tolerance. Further study on the transcriptome or proteome of these genotypes will help to find possible related genes/enzymes with the identified important metabolites. These metabolome data could also be used in future QTL or GWAS studies to identify locus (loci) or gene(s) associated with these metabolic trait(s), and thus to offer gene(s) or trait-specific marker(s) for the crop improvement.

## Figures and Tables

**Table 1:** Statistics of differently accumulated metabolites in each group.

Compared Samples	Num. of Total Ident	Num. of Total Sig	Num. of Sig. Up	Num. of Sig. Down
T1 vs. CK1_pos	3028	527	449	78
T2 vs. CK2_pos	3028	499	397	102
CK2 vs. CK1_pos	3028	811	523	288
T2 vs. T1_pos	3028	797	425	372
T1 vs. CK1_neg	2544	464	366	98
T2 vs. CK2_neg	2544	429	318	111
CK2 vs. CK1_neg	2544	610	390	220
T2 vs. T1_neg	2544	584	326	258

**Table 2:** Identification results of differently accumulated metabolites between two wheat genotypes under drought. FC: fold change, VIP: variable importance in the projection.

Ionization Mode		Classification	Metabolite	RT (min)	m/z	Formula	VIP	FC	*p* Value
ESI (+)	1	Amino acid	L-Isoleucine	1.648	131.0946	C6 H13 N O2	1.51	0.33	1.49 × 10^−7^
ESI (+)	2	Amino acid	L-Tyrosine	1.413	181.0738	C9 H11 N O3	1.40	0.37	1.12 × 10^−5^
ESI (+)	3	Amino acid	L-Phenylalanine	3.199	165.07893	C9 H11 N O2	2.37	0.22	0.014226
ESI (+)	4	Flavonoids	Dhurrin	6.244	311.09967	C14 H17 N O7	1.49	2.97	1.55 × 10^−12^
ESI (+)	5	Flavonoids	Amygdalin	8.234	457.15781	C20 H27 N O11	2.36	0.18	8.67 × 10^−10^
ESI (+)	6	Flavonoids	Luteolin	8.624	286.04691	C15 H10 O6	1.37	0.36	1.83 × 10^−10^
ESI (+)	7	Flavonoids	Salidroside	7.593	300.11993	C14 H20 O7	3.30	0.09	1.41 × 10^−7^
ESI (+)	8	Flavonoids	Quercetin 3-O-sophoroside	7.941	626.14635	C27 H30 O17	1.38	0.43	0.00944
ESI (+)	9	Flavonoids	Arbutin	6.052	272.08903	C12 H16 O7	1.05	0.46	2.43 × 10^−6^
ESI (+)	10	Flavonoids	Keracyanin	10.441	595.1657	C27 H31 O15	1.53	0.32	1.40 × 10^−9^
ESI (+)	11	Alkaloids	N-Methyltryptamine	5.986	174.1156	C11 H14 N2	3.42	0.07	1.98 × 10^−6^
ESI (+)	12	Alkaloids	Harmine	9.559	212.09404	C13 H12 N2 O	2.21	0.20	4.11 × 10^−12^
ESI (+)	13	Alkaloids	Tryptamine	5.638	160.09996	C10 H12 N2	3.98	0.05	1.02 × 10^−8^
ESI (+)	14	Organic acids	Dihydroxyindole	7.901	149.04764	C8 H7 N O2	1.06	2.16	2.82 × 10^−8^
ESI (+)	15	Alkaloids	Indole	5.788	117.05778	C8 H7 N	1.01	0.48	1.32 × 10^−9^
ESI (+)	16	Amino acid	L-Glutamic acid	1.088	147.05307	C5 H9 N O4	1.15	0.43	1.65 × 10^−6^
ESI (+)	17	Phenolics	Vanillin	2.477	152.04724	C8 H8 O3	1.65	3.36	2.90 × 10^−10^
ESI (+)	18	Phenolics	3,4-Dihydroxybenzaldehyde	6.419	138.03162	C7 H6 O3	1.74	3.56	8.94 × 10^−12^
ESI (+)	19	Alkaloids	Tyramine	3.173	137.08408	C8 H11 N O	2.63	0.23	0.045753
ESI (+)	20	Phenolics	3-Dimethylallyl-4-hydroxymandelic acid	8.564	236.1046	C13 H16 O4	1.21	2.43	1.10 × 10^−12^
ESI (+)	21	Alkaloids	Scopoletin	8.437	192.04209	C10 H8 O4	2.26	5.20	5.97 × 10^−12^
ESI (+)	22	Phenolics	Sinapyl alcohol	7.064	210.08902	C11 H14 O4	1.30	0.39	2.92 × 10^−8^
ESI (+)	23	Phenolics	Eugenol	10.704	164.08361	C10 H12 O2	1.02	0.46	1.13 × 10^−5^
ESI (+)	24	Amino acid	L-Cysteinylglycine	6.258	178.04111	C5 H10 N2 O3 S	1.05	2.16	4.38 × 10^−6^
ESI (+)	25	Sugars	D-Ribulose 5-phosphate	1.324	230.01883	C5 H11 O8 P	1.54	3.05	2.43 × 10^−7^
ESI (+)	26	Phenolics	Eriodictyol	10.112	288.0633	C15 H12 O6	2.38	0.18	6.33 × 10^−11^
ESI (+)	27	Others	Farnesyl pyrophosphate	11.295	382.12948	C15 H28 O7 P2	1.10	2.11	0.000214
ESI (+)	28	Others	trans-Zeatin	6.178	219.11171	C10 H13 N5 O	1.15	2.51	0.002331
ESI (+)	29	Pyrimidine	Thymine	4.325	126.04292	C5 H6 N2 O2	3.57	13.72	4.45 × 10^−15^
ESI (+)	30	Amino acid	Fructoselysine	11.044	308.15916	C12 H24 N2 O7	1.45	2.89	1.29 × 10^−8^
ESI (+)	31	Phenolics	Syringic acid	6.276	198.05268	C9 H10 O5	1.55	0.32	6.83 × 10^−11^
ESI (+)	32	Phenolics	alpha-Phocaecholic acid	1.133	424.28289	C24 H40 O6	1.74	3.63	8.48 × 10^−9^
ESI (+)	33	Phenolics	Caffeic acid	10.025	180.04209	C9 H8 O4	1.28	0.42	0.00074
ESI (+)	34	Phenolics	1,4-Dihydroxy-2-naphthoic acid	7.005	204.04184	C11 H8 O4	1.18	0.42	2.88 × 10^−8^
ESI (+)	35	Phenolics	Kynurenic acid	8.003	189.04248	C10 H7 N O3	1.91	3.98	5.60 × 10^−8^
ESI (+)	36	Purine	Guanine	1.934	151.04938	C5 H5 N5 O	1.11	2.34	0.000149
ESI (-)	37	Organic acids	L-Proline	1.165	115.0632	C5 H9 N O2	2.33	0.16	2.24 × 10^−8^
ESI (-)	38	Organic acids	Citric acid	7.876	192.027	C6 H8 O7	1.03	2.31	1.13 × 10^−6^
ESI (-)	39	Organic acids	Fumaric acid	1.029	116.0109	C4 H4 O4	2.11	0.20	2.39 × 10^−6^
ESI (-)	40	Organic acids	trans-Cinnamic acid	2.965	148.0523	C9 H8 O2	1.45	0.31	2.96 × 10^−9^
ESI (-)	41	Phenolics	Ascorbic acid	1.118	176.032	C6 H8 O6	2.30	0.16	9.97 × 10^−8^
ESI (-)	42	Others	Reduced Glutathione	1.066	307.0832	C10 H17 N3 O6 S	1.56	0.28	1.62 × 10^−9^
ESI (-)	43	Phenolics	Homovanillic acid	6.58	182.0578	C9 H10 O4	1.10	2.42	2.62 × 10^−11^
ESI (-)	44	Phenolics	Gallic acid	2.486	170.0215	C7 H6 O5	1.42	3.15	1.87E × 10^−8^
ESI (-)	45	Phenolics	Coniferyl aldehyde	8.86	178.0628	C10 H10 O3	1.57	3.55	2.55 × 10^−9^
ESI (-)	46	Amino acid metabolic intermediates	Saccharopine	0.965	276.1314	C11 H20 N2 O6	1.23	0.39	3.55 × 10^−5^
ESI (-)	47	Phenolics	Gentisic acid	6.882	154.0265	C7 H6 O4	2.80	0.10	1.87 × 10^−9^
ESI (-)	48	Phenolics	Vanillyl alcohol	5.27	154.0629	C8 H10 O3	1.54	3.62	2.76 × 10^−5^
ESI (-)	49	Phenolics	4-Nitrophenol	8.546	139.0268	C6 H5 N O3	1.20	2.63	2.73 × 10^−9^
ESI (-)	50	Phenolics	Pyridoxal	1.622	167.058	C8 H9 N O3	1.11	2.44	1.41 × 10^−8^
ESI (-)	51	Flavonoids	Pumiloside	8.868	512.1788	C26 H28 N2 O9	1.29	0.37	1.59 × 10^−5^
ESI (-)	52	Flavonoids	Genistein	11.175	270.0521	C15 H10 O5	1.15	2.66	0.000446
ESI (-)	53	Flavonoids	Astragalin	8.489	448.1001	C21 H20 O11	1.53	0.31	2.36 × 10^−5^
ESI (-)	54	Flavonoids	Quercitrin	7.573	448.0997	C21 H20 O11	1.89	0.22	1.35 × 10^−9^
ESI (-)	55	Flavonoids	Quercetin 3-O-(beta-D-xylosyl-(1-> 2)-beta-D-glucoside)	6.96	596.1377	C26 H28 O16	1.79	0.24	2.16 × 10^−10^
ESI (-)	56	Phenolics	Picolinic acid	7.886	123.032	C6 H5 N O2	1.17	2.55	5.31 × 10^−11^

## Supplementary Materials

The following are available online at https://www.mdpi.com/2223-7747/9/4/520/s1, Figure S1: Hierarchical cluster heat map of significantly different metabolites between two genotypes after drought stress. (**A**), Positive mode, (**B**), negative mode.
