# Supplementary figures and images for: Metabolomics Response for Drought Stress Tolerance in Chinese Wheat Genotypes (Triticum aestivum)
Source: Plants (Basel). 2020 Apr 17;9(4):520. doi: 10.3390/plants9040520 (PMC7238273; doi:10.3390/plants9040520)

### T2.vs.T1

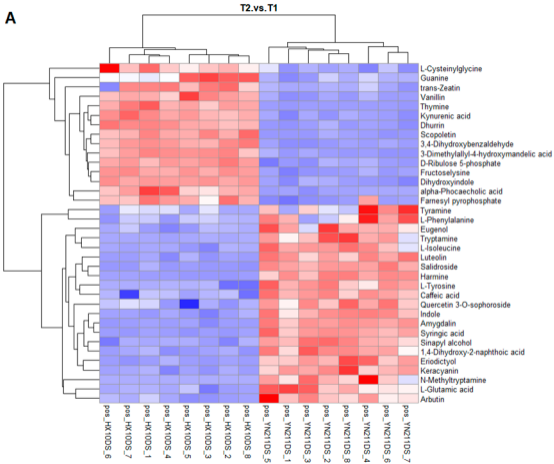

### T2.vs.T1

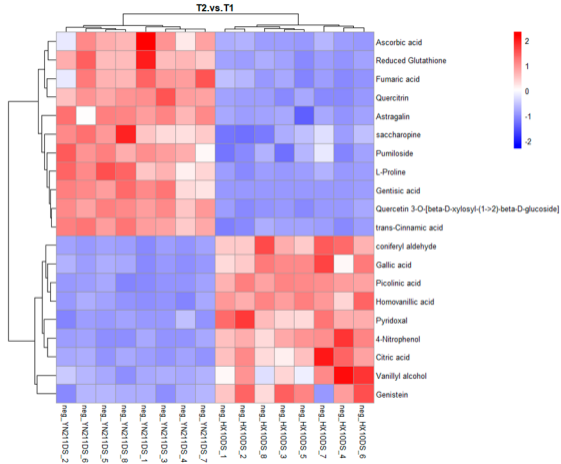

Supplement: Supplementary file 1 [file plants-09-00520-s001.pdf]
